# Soluble Leukocyte-Associated Ig-Like Receptor-1 in Amniotic Fluid Is of Fetal Origin and Positively Associates with Lung Compliance

**DOI:** 10.1371/journal.pone.0083920

**Published:** 2013-12-26

**Authors:** Michiel L. Houben, Marloes J. M. Olde Nordkamp, Peter G. J. Nikkels, Cornelis K. van der Ent, Linde Meyaard, Louis Bont

**Affiliations:** 1 Department of Pediatrics, University Medical Center Utrecht, Utrecht, The Netherlands; 2 Department of Immunology, Laboratory for Translational Immunology, University Medical Center Utrecht, Utrecht, The Netherlands; 3 Department of Pathology, University Medical Center Utrecht, Utrecht, The Netherlands; University of Giessen Lung Center, Germany

## Abstract

The soluble form of the inhibitory immune receptor leukocyte-Associated Ig-like Receptor-1 (sLAIR-1) is present in plasma, urine and synovial fluid and correlates to inflammation. We and others previously showed inflammatory protein expression in normal amniotic fluid at term. We hypothesized that sLAIR-1 is present in amniotic fluid during term parturition and is related to fetal lung function development. sLAIR-1 was detectable in all amniotic fluid samples (n=355) collected during term spontaneous deliveries. First, potential intra-uterine origins of amniotic fluid sLAIR-1 were explored. Although LAIR-1 was expressed on the surface of amniotic fluid neutrophils, LAIR-1 was not secreted upon ex vivo neutrophil stimulation with LPS, or PMA/ionomycin. Cord blood concentrations of sLAIR-1 were fourfold lower than and not related to amniotic fluid concentrations and placentas showed no or only sporadic LAIR-1 positive cells. Similarly, in post-mortem lung tissue of term neonates that died of non-pulmonary disorders LAIR-1 positive cells were absent or only sporadically present. In fetal urine samples, however, sLAIR-1 levels were even higher than in amniotic fluid and correlated with amniotic fluid sLAIR-1 concentrations. Second, the potential relevance of amniotic fluid sLAIR-1 was studied. sLAIR-1 concentrations had low correlation to amniotic fluid cytokines. We measured neonatal lung function in a convenient subset of 152 infants, using the single occlusion technique, at a median age of 34 days (IQR 30-39). The amniotic fluid concentration of sLAIR-1 was independently correlated to airway compliance (ρ=0.29, *P*=.001). Taken together, we show the consistent presence of sLAIR-1 in amniotic fluid, which originates from fetal urine. Concentrations of sLAIR-1 in amniotic fluid during term deliveries are independent from levels of other soluble immune mediators. The positive association between concentrations of amniotic fluid sLAIR-1 and neonatal lung compliance suggests that amniotic fluid sLAIR-1 may be useful as a novel independent marker of neonatal lung maturation.

## Introduction

The immune system plays a pivotal role in the defence of our body by eliminating pathogens on a daily basis. Activation is ensured by pathogen recognition receptors which recognize microbial products such as lipopolysaccharide, flagellin or zymosan[1].To ensure that there is no damage to self by an excess immune response, the immune system needs to be tightly regulated. The expression of inhibitory immune receptors can provide the necessary fine tuning of an immune response[2]. Soluble variants of inhibitory immune receptors may interfere with the function of the membrane bound variant of the receptor.

Leukocyte-associated Ig-like receptor-1 (LAIR-1) was discovered in 1997 as an inhibitory immune receptor that is expressed on multiple peripheral blood leukocytes, including T, B, and NK cells, eosinophils, monocytes and dendritic cells[3,4]. Collagens are ligands for LAIR-1 and cross-linking results in an increase of the threshold for activating signals on several immune cells[5]. Interestingly, its family member LAIR-2 is expressed as a soluble receptor which also binds collagen molecules, and may function as a natural competitor of membrane-bound LAIR-1, serving as a regulator of LAIR-1[6]. sLAIR-1 can also be detected in serum and urine, probably by shedding from the surface of LAIR-1 expressing cells[7]. The affinity of sLAIR-1 for collagen most likely is too low to function as a regulator of LAIR-1 by competing for collagen binding sites[8]. Ouyang and colleagues have demonstrated sLAIR-1 in serum of healthy individuals and increased concentrations in patients with renal disorders[7]. We recently demonstrated that sLAIR-1 levels in urine of rheumatoid arthritis patients are significantly increased as compared to healthy controls. Furthermore, synovial fluid of rheumatoid arthritis patients contained significantly more sLAIR-1 than synovial fluid of osteoarthritis patients, leading to the conclusion that sLAIR-1 is a marker of inflammation[8]. 

In this study, we investigated the levels of sLAIR-1 in amniotic fluid. Intra-uterine inflammation, in absence of a fetal inflammatory response syndrome, promotes fetal lung maturation[9-12]. In a cohort of 761 preterm infants, chorioamnionitis protected against the development of chronic lung disease (CLD)[13]. Moreover, in preterm lambs, experimental intra-amniotic injection of bacterial endotoxin induced an inflammatory response in membranes and in amniotic fluid, resulting in increased fetal lung compliance and lung volume[9,11,12,14]. The mechanisms underlying the beneficial effects of intra-uterine inflammation on fetal lung maturation have not yet been identified.

We hypothesized that sLAIR-1 is present in utero during term spontaneous onset of labor delivery and reflects a general state of immune activation[15,16]. Subsequently, we studied whether intra-amniotic sLAIR-1 is a marker of normal fetal lung function in a large healthy birth cohort.

## Methods

### Study population and baseline characteristics

This study was performed as part of the Netherlands Amniotic Fluid study, described previously[17,18]. In short, 372 healthy term newborns were included in a birth cohort. See Table **S1** for baseline and clinical characteristics.

### Ethics statement

The study protocol was approved by the institutional review boards of the University Medical Center Utrecht and the Diakonessen Hospital (both in Utrecht, The Netherlands) and written informed consent was provided by the parents of all participating children. This provision was waived by the board of the University Medical Center Utrecht in the three (retrospective) cases of pathology of lung tissue of fatal neonatal cases.

### Collection of amniotic fluid, urine, cord blood plasma, placentas and newborn lung tissue

Amniotic fluid was sampled during labor, purified, and stored (-80°C), as described previously[17]. Amniotic fluid cell immunophenotyping and stimulation were performed on fresh amniotic fluid samples. First newborn urine (i.e. fetal urine) was collected from boys, using a urine collection bag, and stored at -80°C. Cord blood was collected directly after birth and anticoagulated using sodium heparin. Plasma was prepared by centrifugation (5 min, 500 *g*), and stored at -80°C. Placentas were stored at +4°C and processed within 72 hours, as described previously[17]. Amniotic fluid lecithin-sphingomyelin (L/S) ratios were determined by thin layer chromatography according to Gluck and Kulovich[19,20]. Representative sections of both lungs of fatal neonatal cases were stored according to standard operating procedures. Three cases were selected retrospectively from a coded database of perinatal autopsies, kept by the pediatric pathologist. The selection was based on delivery at term and on the absence of any signs of pulmonary disorders from the history, physical examination, and macroscopy and microscopy at autopsy.

### sLAIR-1 ELISA

The concentration of sLAIR-1 in amniotic fluid, in cord blood plasma, in fetal urine, and in supernatant of stimulated amniotic fluid cells was measured by sandwich ELISA (in-house manufactured; limit of detection 1.95 ng/mL)[8]. The intra-assay and the inter-assay correlations were high (Spearman’s ρ= 0.98, *P*= .005, *n*=5; Pearson’s ρ= 0.81, *P*< .001, *n*=21).

### Amniotic fluid cytokine and chemokine measurements

In a subsample of 42 amniotic fluid samples, the concentrations of IL-1β, IL-5, IL-6, IL-8, IL-10, IL-12p70, IL-17, IL-18, IL-23, TNF-α, MCP-1, MIF, sICAM, MIP-1α, eotaxin, IP-10, and MIG were measured using ELISA (IL-6, IL-8, and TNF-α; CLB, Sanquin, Amsterdam, The Netherlands)[17] or Luminex[21]. Correlations between these cytokines and amniotic fluid sLAIR-1 were calculated.

### Immunophenotyping of amniotic fluid neutrophils

Amniotic fluid samples were stained with PE-labelled α-hLAIR-1, APC labelled α-CD11b, Pacific Blue labelled α-CD16 and APC-Cy7 labelled α-CD14 antibodies. IgG1 isotype controls were carried out using PE labelled mouse IgG1κ, to rule out non-specific binding (all antibodies were purchased from BD Pharmingen). Neutrophils were selected from the live cell gate of the forward-sideward scatter plot as CD11b^+^ / CD16^+^ / CD14^-^ cells. Macrophages were selected accordingly as CD14^+^ / CD16^-^ cells. Flow cytometry was performed using a LSRII flow cytometer (BD Biosciences, San Diego, CA). Data were analyzed using FlowJo version 7.6 software (Tree Star, Ashland, USA).

### Stimulation of amniotic fluid cells

Fresh amniotic fluid was filtered twice through a 70 µm filter. Cells were isolated by centrifugation, and cultured in RPMI 1640 (Gibco, Invitrogen, The Netherlands) supplemented with 10% fetal calf serum (Integro, Dieren, the Netherlands) and antibiotics at 37°C, 5% CO_2_. Cells were stimulated with LPS (1 ng/mL and 10 ng/mL), and PMA (50 ng/mL) and ionomycin (1 μM) for 24 hours. The supernatant was harvested and stored at -20°C until further use in a sandwich ELISA for the presence of sLAIR-1.

### Placenta and newborn lung immunohistology

Placenta and newborn lung tissue was analyzed for the presence of membrane-bound LAIR-1. Two sections of the umbilical cord, at the fetal and placental side, a membrane roll, one sample from the umbilical cord insertion, and three slides of normal placental parenchyma, including both decidua and chorionic plate and representative sections of newborn lung specimens were collected.

The samples were embedded in paraffin wax by standard histological procedures. Histological sections were cut at 3-4 um and were mounted on coated slides. For all immunohistochemical staining, the same antigen-retrieval method was followed. Before staining with antibodies, slides were placed in boiling citrate buffer (pH 6.0) for 20 min. Monoclonal mouse antibodies against CD68/ED1 (Novo Castra, Newcastle, UK) were used to determine the presence of macrophages. For the placenta slides staining was visualized using vectastain ABC (Vector Laboratories) and diaminobenzidine. For the lung slides staining was visualized using the Bond-max (Leica) automatic stainer using polymer refine red detection. Haematoxylin was used as counterstaining.

To differentiate between cells of trophoblastic (fetal) and decidual (maternal) origin, the samples were stained for the presence of keratin (positive in trophoblast). Histological chorioamnionitis was diagnosed based on the presence of polymorphonuclear cells (neutrophilic granulocytes) in the chorionic plate or the extraplacental membranes[17]. Pharyngeal tonsillary tissue from regular pediatric tonsillectomy specimens was used as positive control tissue for LAIR-1 staining. No negative controls were tested.

### Infant lung function

Infant lung function was measured before the age of two months (median age 34 days, IQR 30-39), during natural sleep and without the use of any sedation, as described previously[22-24]. Lung function was assessed from measurement of passive respiratory mechanics (compliance and resistance of the total respiratory system) using the single occlusion technique (SOT). The results of compliance and resistance were standardized by correction for length, weight and age during lung function measurement.

### Clinical definitions

Antepartum exposure to tobacco smoke was defined as maternal smoking of at least one cigarette per day during the second semester of pregnancy. Parental atopy was defined as the presence of any atopic diagnosis (asthma, eczema or hay fever) made by a physician in one or both parents. Parental asthma was defined accordingly[25].

### Statistical analysis

Baseline and lung function characteristics were compared between groups using Student’s T test, Mann-Whitney U test or X^2^ test, as appropriate. After logarithmic transformation, the concentration of amniotic fluid sLAIR-1 was normally distributed. To assess the association between the amniotic fluid concentration of sLAIR-1 and the standardized airway compliance and resistance, Pearson’s correlation coefficient was calculated. Linear regression analysis was carried out to adjust for baseline characteristics. Spearman’s correlation was calculated for the association with L/S ratios, and the correlation of cytokine and chemokine measurements.

## Results

### sLAIR-1 is detectable in all amniotic fluid samples of term deliveries

**Figure 1:**
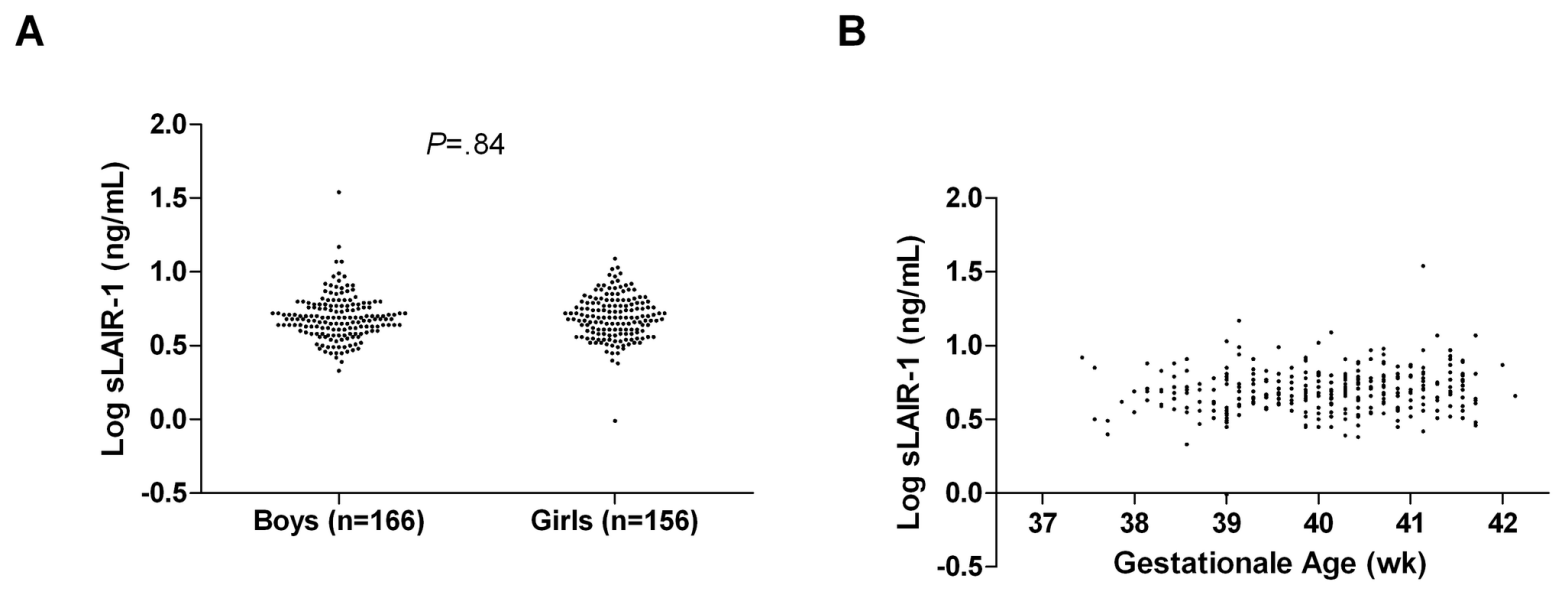
Distribution of amniotic fluid sLAIR-1 during term deliveries. Amniotic fluid was collected transvaginally during term physiologic deliveries (n=345) as described previously(17). sLAIR-1 was measured by sandwich ELISA (limit of detection 1.95 ng/mL). (**A**) Amniotic sLAIR-1 concentration of newborn boys and girls. (**B**) Amniotic sLAIR-1 concentration plotted against gestational age.

The mean concentration of amniotic fluid sLAIR-1 was 4.82 ng/mL (95% CI 2.5-9.4), comparable to the levels of sLAIR-1 found in synovial fluid of ostheoarthritis patients, plasma in rheumatoid arthritis patients, and urine in healthy controls[8]. sLAIR-1 was detectable in all 355 amniotic fluid samples. There were no differences detected for gender, gestational age (Figure **1A-B**), parental asthma, or exposure to maternal tobacco smoking during pregnancy (data not shown). 

### Amniotic fluid neutrophils express LAIR-*1* but do not secrete sLAIR-1 upon stimulation

**Figure 2:**
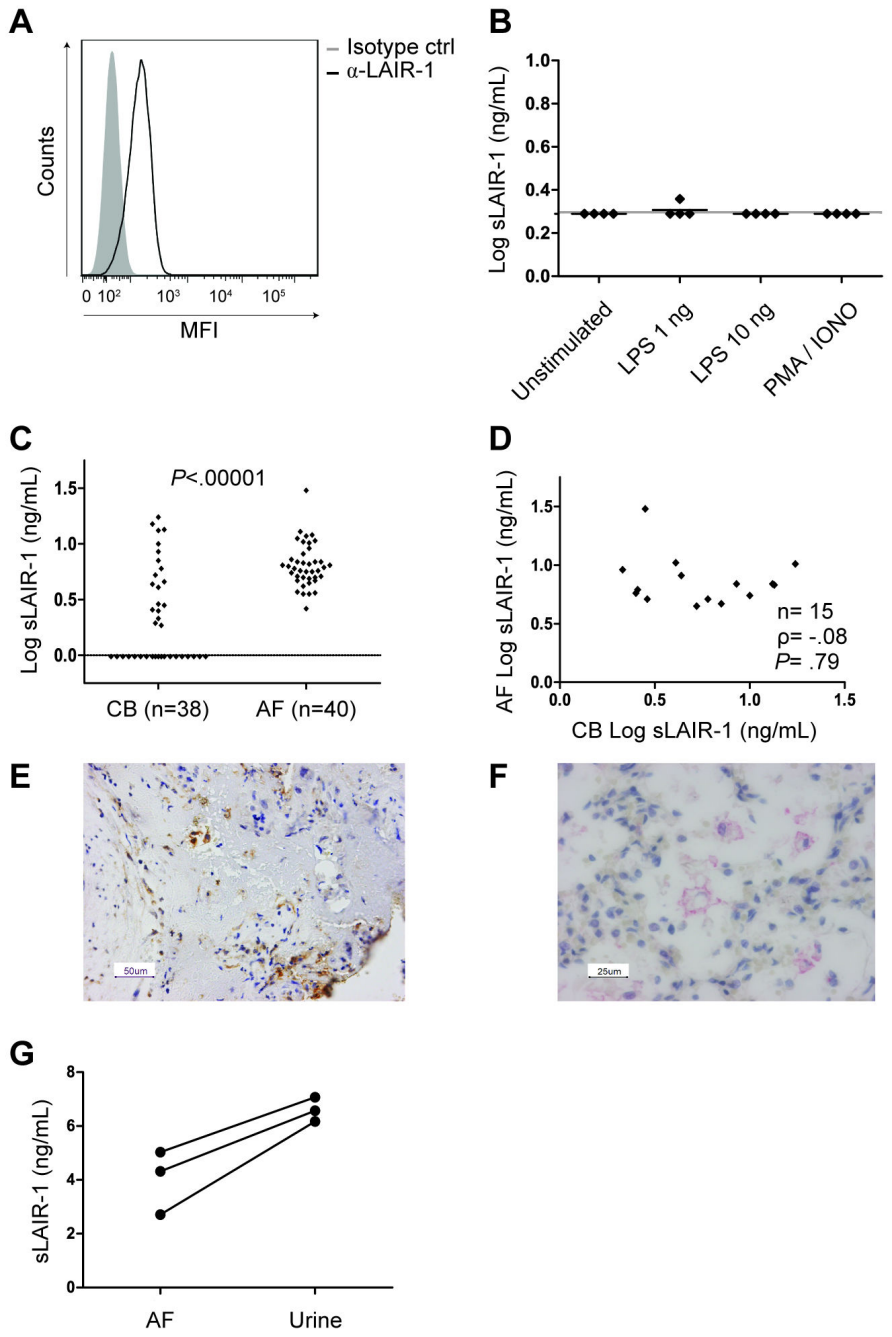
Fetal urine is the source of amniotic fluid sLAIR-1. The origin of amniotic fluid sLAIR-1 was investigated in multiple compartments. (**A**) LAIR-1 flow cytometry on fresh amniotic fluid samples. Neutrophils were selected from the live cell gate of the forward-sideward scatter plot as CD11b^+^ / CD16^+^ / CD14^-^ cells. LAIR-1 expression was measured (representative of a series of *n*=5). (**B**) Amniotic fluid cells were stimulated with LPS or PMA / ionomycin (*n*=4). After 24 hours supernatant was harvested and the concentration of sLAIR-1 was measured in a sandwich ELISA. All values below the limit of detection were plotted as the limit of detection. Notably, flow cytometry did not consistently demonstrate the expression of LAIR-1 on the cell surface of amniotic fluid macrophages (data not shown). (**C**) sLAIR-1 ELISA in samples of cord blood (CB) and amniotic fluid (AF). Comparison of the concentration of sLAIR-1 in CB and AF samples. Student’s T test for unpaired analysis. (**D**) Correlation between detectable sLAIR-1 in paired cord blood and amniotic fluid samples (Pearson’s correlation coefficient). (**E**) Immunohistology of a placenta with signs of severe chorioamnionitis. LAIR-1 positive cells are infrequently present in the chorionic plate. (**F**) Immunohistology of lung tissue of a child that died perinatally of an urea cycle defect. Lung microscopy without signs of inflammation and negative LAIR-1 staining, except for sporadic interstitial cells and for alveolary macrophages. (**G**) sLAIR-1 ELISA in paired samples of amniotic fluid (AF) and fetal urine.

We sought to identify the intra-uterine origin of amniotic fluid sLAIR-1. LAIR-1 was expressed on the cell surface of neutrophils in amniotic fluid, as assessed by flow cytometry (Figure **2A**). However, upon ex vivo culture and stimulation of isolated amniotic fluid cells with LPS or PMA / ionomycin, no secretion of sLAIR-1 in supernatant was detected (Figure **2B**). Previous studies have revealed the presence of macrophages in amniotic fluid at term, next to neutrophil abundancy[17]. Flow cytometry did not consistently demonstrate the expression of LAIR-1 on the cell surface of amniotic fluid macrophages (data not shown).

### Amniotic fluid sLAIR-1 is not related to cord blood sLAIR-1

Next, we measured the concentration of sLAIR-1 in paired cord blood and amniotic fluid samples, to assess for spill-over of sLAIR-1 from cord blood to amniotic fluid. sLAIR-1 was detected in 50% of cord blood plasma samples versus 100% of amniotic fluid samples (Figure **2C**). There was no correlation between the concentrations of sLAIR-1 in paired samples of cord blood plasma (with detectable sLAIR-1) and amniotic fluid (Figure **2D**).

### No correlation between amniotic fluid sLAIR-1 and L/S ratio

Lecithin and sphingomyelin are surfactant glycoproteins and their ratio is used to predict preterm lung maturation. Amniotic fluid L/S ratios were determined in 21 amniotic fluid samples to assess the relation to surfactant synthesis at term. The mean L/S ratio was 9.2 (SD 4.1, range 3.1 to 18.2). Amniotic fluid L/S ratios were not correlated to the amniotic fluid concentrations of sLAIR-1 (Spearman’s ρ=0.02, *P*=.94, *n*=21). In addition, L/S ratios were not associated with amniotic fluid pro-inflammatory cytokine concentrations, airway compliance or resistance, or gestational age or birth weight (data not shown).

### No LAIR-1 positive cells present in placenta tissue during spontaneous onset of labor at term

The placentas of three children were analyzed. The two placentas without or with mild signs of histological chorioamnionitis were LAIR-1 negative (Figure **S1** and Figure **S2**). The placenta with signs of severe chorioamnionitis showed infrequent positive LAIR-1 staining of cells in the chorionic plate, stromal cells in Wharton’s jelly of the umbilical cord, and the maternal side of the chorionic membranes (Figure **2E**, Figure **S3**). Within the chorionic membranes, LAIR-1 was only detectable in regions that stained negative for keratin, suggesting a maternal origin of the LAIR-1 positive cells. LAIR-1 was predominantly located on the membrane of cells, most consistently in the keratin negative regions (Figure **S4**). Cells that stained LAIR-1 positive were mononuclear on microscopy and stained CD68 negative (data not shown). Tonsillary tissue (positive control) stained positive for LAIR-1 (data not shown).

### No LAIR-1 positive cells present in lung tissue of fatal perinatal cases

We analyzed lung tissue of three children that died perinatally without evidence of any pulmonary disorder. The first case was a girl born after a gestational age of 37 weeks, who died from perinatal asphyxia. Lung microscopy showed sporadic infiltrates of neutrophils. Staining for LAIR-1 was negative (data not shown). The second case was a boy born after a gestational age of 37 weeks, who died from multiple congenital anomalies, including ventricular septal defect, schizencephaly, hydrocephalus and corpus callosum agenesis. Lung microscopy showed no abnormalities, except for weak LAIR-1 staining in the interstitium (Figure **S5**). The third case was a boy born after a gestational age of 38 weeks, who developed hypothermia, convulsions and severe hyperammonemia on the second day of life. He died under the diagnosis of an urea cycle disorder. Lung microscopy showed no signs of inflammation and LAIR-1 staining was negative, except for alveolar macrophages (Figure **2F**, Figure **S6**). In summary, LAIR-1 was demonstrated to be virtually absent in term fetal lung parenchyma and epithelial tissue. In some cases alveolar macrophages stained positive.

### Urine is the fetal source of sLAIR in amniotic fluid

Finally, we studied whether sLAIR is introduced in the amniotic cavity by fetal urine, because amniotic fluid is mainly produced by fetal urine. Indeed, we found high concentrations of sLAIR-1 in fetal urine samples which exceeded levels of paired amniotic fluids samples (Figure **2G**). We therefore conclude that the sLAIR-1 in amniotic fluid is of fetal origin.

### Infant lung function is associated with the concentration of amniotic fluid sLAIR-1

We hypothesized that the abundant level of sLAIR-1 in amniotic fluid could relate to a positive effect on the infant lung development. Lung function testing of all newborns was carried out, using the single occlusion technique. Successful lung function measurement was performed in 152 infants. The major reasons for failure of lung function measurement were: technical (insufficient quality and / or number of occlusions, 42%) and no sleep or too short period of sleep (52%). Children with successful lung function measurements and those who failed had similar baseline characteristics (Table **S1**). Compliance and resistance of the respiratory system were normally distributed after logarithmic transformation (Figure **S7**).

**Figure 3:**
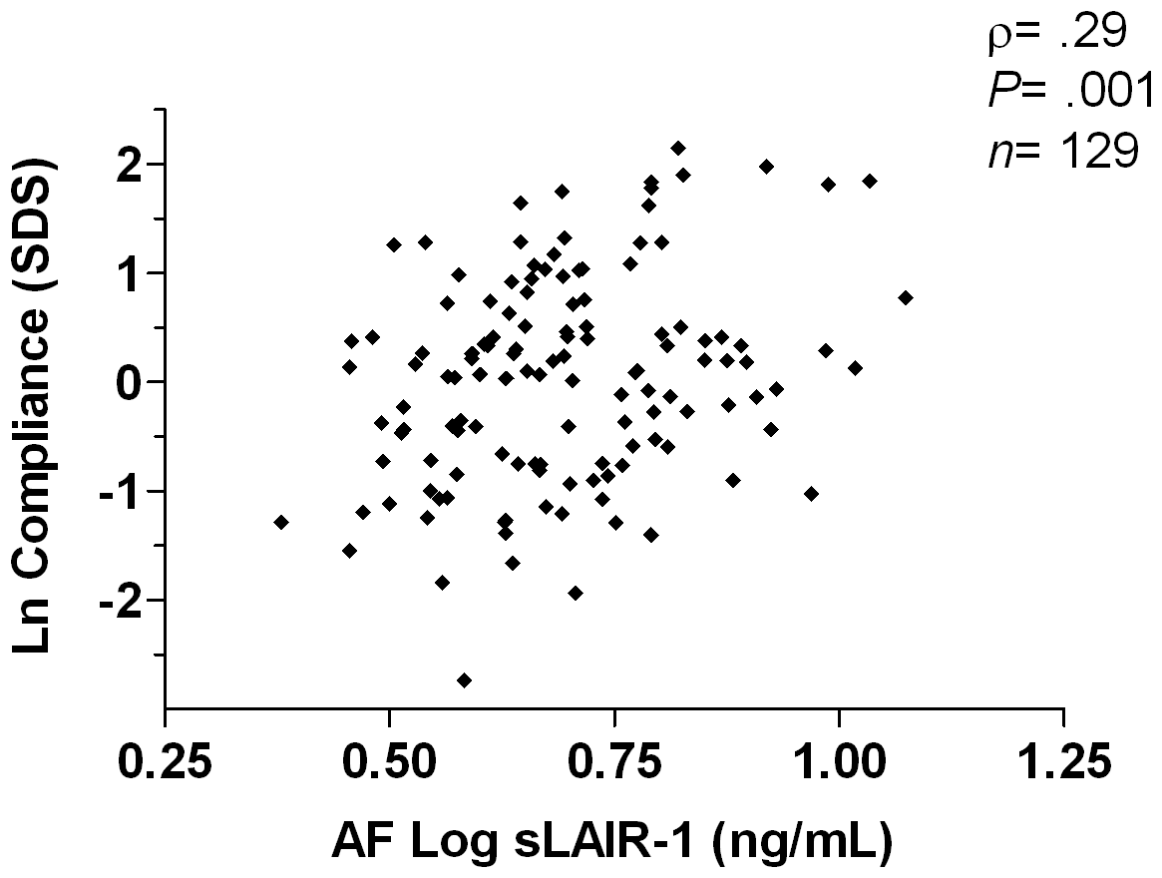
Correlation between amniotic fluid sLAIR-1 and infant lung compliance. Amniotic fluid sLAIR-1 was measured by sandwich ELISA (limit of detection 1.95 ng/mL). Infant lung compliance was assessed using the single occlusion technique during physiologic sleep. Compliance measurements were standardized, correcting for length, weight, and age during lung function measurement. Pearson’s correlation was calculated.

There was a moderately strong, positive association between the amniotic fluid concentration of sLAIR-1 and infant lung compliance (ρ=0.29, *P*=.001, *n*=129, Figure **3**). Adjustment for sex yielded identical results (ρ=0.28, *P*=.002, *n*=129; girls ρ=0.23, *P*=.07, *n*=63; boys ρ=0.33, *P*=.006, *n*=66). Adjustment for antepartum maternal smoking yielded similar results (correction: ρ=0.28, *P*=.001, *n*=129; no smoking ρ=0.28, *P*=.002, *n*=113). There was no association between the amniotic fluid concentration of sLAIR-1 and infant resistance of the respiratory system (ρ= -0.08, *P*=.37, *n*=131).

### Amniotic sLAIR-1 is a distinct marker for fetal lung development

To study whether this relation is specific for amniotic fluid sLAIR-1, we measured a series of established (mainly) pro-inflammatory cytokines and chemokines in a subsample of the cohort. We found high correlations between amniotic fluid concentrations of IL-1β, IL-5, IL-6, IL-8, IL-10, IL-12p70, IL-17, IL-18, IL-23, TNF-α, MCP-1, MIF, sICAM, MIP-1α, eotaxin, IP-10, and MIG, (Table **S2**), resembling the “acute inflammation gene expression signature” described by Haddad and colleagues[15]. Interestingly, the pattern of amniotic fluid sLAIR-1 concentration was distinct and poorly associated with the before mentioned cytokines. The effect of sLAIR-1 on infant lung function was not found for other pro-inflammatory cytokines nor for placenta histopathology (data not shown). Thus amniotic fluid sLAIR-1 is a distinct marker for fetal lung development.

## Discussion

In a birth cohort of healthy term newborns, we demonstrated the consistent presence of sLAIR-1 in amniotic fluid. Concentrations were independent from amniotic fluid cytokine levels. Clinical evaluation at age one month of these newborns showed an association between intra-amniotic sLAIR-1 and newborn lung function. We found a positive correlation (ρ=0.29) between the amniotic fluid concentration of sLAIR-1 and compliance of the total respiratory system. High levels of sLAIR-1 in paired fetal urine and amniotic fluid samples showed that fetal urine is the most likely source of amniotic fluid sLAIR-1.

The number of studies on sLAIR-1 is limited. Previous studies show that sLAIR-1 reflects a state of immune activation, and might even be used as a biomarker for disease activity[7,8,26]. It was hypothesized that LAIR-1 is shed from the cell membrane of activated lymphocytes, possibly blocking the interaction with its ligand, thereby allowing for persistent lymphocyte activation[4,7]. However, we recently showed that the affinity of sLAIR-1 is too low to function as a competitor for ligand binding sites[8]. Still, when shed of the cell membrane, LAIR-1 expression on the cell surface is decreased, and thereby the inhibitory signal is diminished. We think that amniotic fluid sLAIR-1 reflects perinatal in utero immune activation[15,16]. Immune activation could contribute to increased lung maturation at term and amniotic fluid sLAIR-1 could be either a direct signal for lung maturation, or, more likely, a reflection of the fetal inflammatory state.

Experimental studies in animals and observational studies in humans support the suggestion of a (complex) relation between fetal lung maturation, intra-amniotic inflammation and sLAIR-1. Intra-amniotic induction of a pro-inflammatory response doubled lung compliance and lung volume in prematurely delivered lambs[11,14]. Similarly, intra-amniotic injection of interleukin-1β (IL-1β), TNF-α, IL-6, and IL-8 in pregnant rhesus monkeys induced accumulation of neutrophils in the fetal lungs[27]. Studies on the relation between intra-amniotic inflammation and lung function in term newborns are lacking. Haddad and colleagues showed that an “acute inflammation gene expression signature” is generally present during physiologic term deliveries, and it was speculated that tissue homeostasis could be promoted by intra-uterine inflammation[15]. Previously, we have demonstrated that extensive intra-uterine inflammation is present during term delivery with spontaneous onset of labor[17]. Remarkably, we found that amniotic fluid sLAIR-1 levels are independent of the levels of other pro-inflammatory cytokines, and that only sLAIR-1 correlates to newborn lung function. Conceivably, sLAIR-1 is a unique representative of tissue inflammation or cell activation with limited overlap with pro-inflammatory cytokines, such as TNF-α or IL-1-β. 

A limitation of our study is the observational nature. The complex relation between intra-amniotic inflammation, amniotic fluid sLAIR-1 and infant lung function remains to be unraveled. The major strength of our study is that we had the opportunity to study sLAIR-1 in a large cohort of healthy term deliveries and that several intra-uterine samples were available for extensive assessment.

Our study potentially has clinical implications. The association between intra-uterine sLAIR-1 and infant lung function may extend our insight on the origin of childhood respiratory disorders[28-32]. Furthermore, it increases our insight in the mechanisms underlying fetal and newborn lung development[11,14,33]. In future, amniotic fluid proteins such as sLAIR-1 possibly may serve as biomarkers for early detection of the susceptibility to viral LRTIs or recurrent wheeze[34-36]. Eventually, such a biomarker can also be applied to target new preventive or treatment strategies to children at high risk of respiratory syncytial virus [RSV] or asthma development[37,38].

In conclusion, infant lung compliance is positively correlated with the amniotic fluid concentration of sLAIR-1 in healthy term infants. Fetal urine is the source of amniotic fluid sLAIR-1. The high level of amniotic fluid sLAIR-1 during term parturition probably reflects a general state of perinatal immune activation and may be a biomarker of lung maturation in term fetuses. These novel findings may improve our understanding of the origins of childhood respiratory disorders in general, and of the physiologic maturation of fetal and infant lung function specifically.

## Supporting Information

Figure S1 **No LAIR-1 positive cells present in placenta tissue during spontaneous onset of labor at term: Placenta without histological signs of chorioamnionitis.** Immunohistology of placenta. Standard H/E staining was applied to samples without immunohistology staining. LAIR-1 positive cells are absent in the chorionic plate (1+2), the umbilical cord, and the chorionic membranes (data not shown).
(ZIP)

Figure S2 **No LAIR-1 positive cells present in placenta tissue during spontaneous onset of labor at term: Placenta with mild signs of chorioamnionitis.** Immunohistology of placenta. Standard H/E staining was applied to samples without immunohistology staining. LAIR-1 positive cells are sparsely present in the chorionic membranes (1+2+4), the stromal cells of Wharton’s jelly of the umbilical cord (3), and the chorionic plate (data not shown).
(ZIP)

Figure S3 **No LAIR-1 positive cells present in placenta tissue during spontaneous onset of labor at term: Placenta with severe signs of chorioamnionitis.** Immunohistology of placenta. Standard H/E staining was applied to samples without immunohistology staining. LAIR-1 positive cells are infrequently present in the chorionic plate (see Figure 2E), the stromal cells of Wharton’s jelly of the umbilical cord (1), and the maternal side of the chorionic membranes (2+3).
(ZIP)

Figure S4 **No LAIR-1 positive cells present in placenta tissue during spontaneous onset of labor at term: Placenta with severe signs of chorioamnionitis.** Immunohistology of placenta. Standard H/E staining was applied to samples without immunohistology staining. LAIR-1 (left, 1+3) and keratin (right, 2+4) immunohistology. LAIR-1 positive cells are present in keratin negative regions (decidua) of the chorionic membranes. On these mononuclear cells, LAIR-1 staining is predominantly found on the cell membrane. CD68 staining (macrophage marker) of these cells is negative (data not shown). LAIR-1 positive cells are sparsely present in keratin positive regions (trophoblast) of chorionic membranes.
(ZIP)

Figure S5 **No LAIR-1 positive epithelial cells present in lung tissue of second term fatal neonatal case.** Immunohistology of lung tissue of a child (second case) that died perinatally without evidence of any pulmonary disorder. Lung microscopy without abnormalities, except for weak positive LAIR-1 staining interstitially (1+2).
(ZIP)

Figure S6 **No LAIR-1 positive epithelial cells present in lung tissue of third term fatal neonatal case.** Immunohistology of lung tissue of a child (third case) that died perinatally without evidence of any pulmonary disorder. Lung microscopy without signs of inflammation and negative LAIR-1 staining, except for alveolar macrophages (1-3).
(ZIP)

Figure S7 **Compliance and resistance of the respiratory system of healthy term newborns.** Newborn lung compliance (left Y-axis) and resistance (right Y-axis) were assessed using the single occlusion technique during physiologic sleep. Mean (standard deviation) compliance 47.8 (11.3) mL/kPa. Mean (standard deviation) resistance 6.27 (1.7) kPa/L/s.
(ZIP)

Table S1 **Baseline characteristics.** Values represent mean (SD) or percentage. *P*-values for Student’s T test or X^2^ test. Missing values: parental atopy and / or asthma *n*=7 (2%), maternal antepartum smoking *n*=13 (3%). NA denotes not applicable.
(DOC)

Table S2 **Amniotic fluid cytokine and chemokine correlation matrix.** Spearman’s ρ (upper) and *P*-value (lower). Sample sizes were 40-42.
(DOC)

## References

[1] Akira S, Uematsu S, Takeuchi O (2006) Pathogen recognition and innate immunity. Cell 124(4): 783-801. doi:10.1016/j.cell.2006.02.015. PubMed: 16497588. 16497588

[2] Ravetch JV, Lanier LL (2000) Immune inhibitory receptors. Science 290(5489): 84-89. doi:10.1126/science.290.5489.84. PubMed: 11021804. 11021804

[3] Meyaard L, Adema GJ, Chang C, Woollatt E, Sutherland GR et al. (1997) LAIR-1, a novel inhibitory receptor expressed on human mononuclear leukocytes. Immunity 7(2): 283-290. doi:10.1016/S1074-7613(00)80530-0. PubMed: 9285412. 9285412

[4] Meyaard L (2008) The inhibitory collagen receptor LAIR-1 (CD305). J Leukoc Biol 83(4): 799-803. doi:10.1189/jlb.0907609. PubMed: 18063695. 18063695

[5] Lebbink RJ, de Ruiter T, Adelmeijer J, Brenkman AB, van Helvoort JM et al. (2006) Collagens are functional, high affinity ligands for the inhibitory immune receptor LAIR-1. J Exp Med 203(6): 1419-1425. doi:10.1084/jem.20052554. PubMed: 16754721. 16754721 PMC2118306

[6] Lebbink RJ, van den Berg MC, de Ruiter T, Raynal N, van Roon JA et al. (2008) The soluble leukocyte-associated Ig-like receptor (LAIR)-2 antagonizes the collagen/LAIR-1 inhibitory immune interaction. J Immunol 180(3): 1662-1669. PubMed: 18209062. 18209062 10.4049/jimmunol.180.3.1662

[7] Ouyang W, Xue J, Liu J, Jia W, Li Z et al. (2004) Establishment of an ELISA system for determining soluble LAIR-1 levels in sera of patients with HFRS and kidney transplant. J Immunol Methods 292(1-2): 109-117. doi:10.1016/j.jim.2004.06.005. PubMed: 15350516. 15350516

[8] Olde Nordkamp MJ, van Roon JA, Douwes M, de Ruiter T, Urbanus RT, Meyaard L (2011) Enhanced secretion of leukocyte-associated immunoglobulin-like receptor 2 (LAIR-2) and soluble LAIR-1 in rheumatoid arthritis: LAIR-2 is a more efficient antagonist of the LAIR-1-collagen inhibitory interaction than is soluble LAIR-1. Arthritis Rheum 63(12): 3749-3757. doi:10.1002/art.30612. PubMed: 22127695. 22127695

[9] Kallapur SG, Kramer BW, Nitsos I, Pillow JJ, Collins JJ et al. (2011) Pulmonary and systemic inflammatory responses to intra-amniotic IL-1alpha in fetal sheep. Am J Physiol Lung Cell Mol Physiol 301(3): L285-L295. doi:10.1152/ajplung.00446.2010. PubMed: 21665964. 21665964 PMC3174746

[10] Kramer BW, Jobe AH (2005) The clever fetus: responding to inflammation to minimize lung injury. Biol Neonate 88(3): 202-207. doi:10.1159/000087583. PubMed: 16210842. 16210842

[11] Newnham JP, Moss TJ, Kramer BW, Nitsos I, Ikegami M et al. (2002) The fetal maturational and inflammatory responses to different routes of endotoxin infusion in sheep. Am J Obstet Gynecol 186(5): 1062-1068. doi:10.1067/mob.2002.122293. PubMed: 12015538. 12015538

[12] Willet KE, Kramer BW, Kallapur SG, Ikegami M, Newnham JP et al. (2002) Intra-amniotic injection of IL-1 induces inflammation and maturation in fetal sheep lung. Am J Physiol Lung Cell Mol Physiol 282(3): L411-L420. PubMed: 11839534. 11839534 10.1152/ajplung.00097.2001

[13] Lahra MM, Beeby PJ, Jeffery HE (2009) Intrauterine inflammation, neonatal sepsis, and chronic lung disease: a 13-year hospital cohort study. Pediatrics 123(5): 1314-1319. doi:10.1542/peds.2008-0656. PubMed: 19403497. 19403497

[14] Kramer BW, Moss TJ, Willet KE, Newnham JP, Sly PD et al. (2001) Dose and time response after intraamniotic endotoxin in preterm lambs. Am J Respir Crit Care Med 164(6): 982-988. doi:10.1164/ajrccm.164.6.2103061. PubMed: 11587983. 11587983

[15] Haddad R, Tromp G, Kuivaniemi H, Chaiworapongsa T, Kim YM et al. (2006) Human spontaneous labor without histologic chorioamnionitis is characterized by an acute inflammation gene expression signature. Am J Obstet Gynecol 195(2): 394-324. doi:10.1016/j.ajog.2005.08.057. PubMed: 16890549. 16890549 PMC1800883

[16] Smith R (2007) Parturition. N Engl J Med 356(3): 271-283. doi:10.1056/NEJMra061360. PubMed: 17229954. 17229954

[17] Houben ML, Nikkels PG, van Bleek GM, Visser GHA, Rovers MM et al. (2009) The association between intrauterine inflammation and spontaneous vaginal delivery at term: a cross-sectional study. PLOS ONE 4(8): e6572. doi:10.1371/journal.pone.0006572. PubMed: 19668329. 19668329 PMC2718580

[18] Houben ML, Rovers MM, Wilbrink B, Belderbos ME, Bloemen-Carlier EM et al. (2012) High concentrations of amniotic fluid proinflammatory cytokines in healthy neonates are associated with low risk of respiratory syncytial virus bronchiolitis. Pediatr Infect Dis J 31(9): 931-934. doi:10.1097/INF.0b013e31826366e3. PubMed: 22699404. 22699404

[19] Gluck L, Kulovich MV (1973) Lecithin-sphingomyelin ratios in amniotic fluid in normal and abnormal pregnancy. Am J Obstet Gynecol 115(4): 539-546. PubMed: 4739313. 4739313 10.1016/0002-9378(73)90404-3

[20] Wijnberger LD, de Kleine M, Voorbij HA, Arabin B, van de Leur JJ et al. (2003) The effect of clinical characteristics on the lecithin/sphingomyelin ratio and lamellar body count: a cross-sectional study. J Matern Fetal Neonatal Med 14(6): 373-382. doi:10.1080/14767050412331312210. PubMed: 15061315. 15061315

[21] de Jager W, te VH, Prakken BJ, Kuis W, Rijkers GT (2003) Simultaneous detection of 15 human cytokines in a single sample of stimulated peripheral blood mononuclear cells. Clin Diagn Lab Immunol 10(1): 133-139. PubMed: 12522051. 12522051 10.1128/CDLI.10.1.133-139.2003 PMC145264

[22] Katier N, Uiterwaal CS, de Jong BM, Kimpen JL, van der Ent CK (2005) Feasibility and variability of neonatal and infant lung function measurement using the single occlusion technique. Chest 128(3): 1822-1829. doi:10.1378/chest.128.3.1822. PubMed: 16162792. 16162792

[23] Katier N, Uiterwaal CS, de Jong BM, Verheij TJ, van der Ent CK (2006) Passive respiratory mechanics measured during natural sleep in healthy term neonates and infants up to 8 weeks of life. Pediatr Pulmonol 41(11): 1058-1064. doi:10.1002/ppul.20492. PubMed: 16998930. 16998930

[24] Lesouef PN, England SJ, Bryan AC (1984) Passive respiratory mechanics in newborns and children. Am Rev Respir Dis 129(4): 552-556. PubMed: 6711998. 6711998

[25] Lakwijk N, Van Strien RT, Doekes G, Brunekreef B, Gerritsen J. (1998) (19998) Validation of a screening questionnaire for atopy with serum IgE tests in a population of pregnant Dutch women. Clin Exp Allergy 28(4): 454-458. PubMed: 9641572. 9641572 10.1046/j.1365-2222.1998.00254.x

[26] Lebbink RJ, Meyaard L (2007) Non-MHC ligands for inhibitory immune receptors: novel insights and implications for immune regulation. Mol Immunol 44(9): 2153-2164. doi:10.1016/j.molimm.2006.11.014. PubMed: 17188357. 17188357

[27] Sadowsky DW, Adams KM, Gravett MG, Witkin SS, Novy MJ (2006) Preterm labor is induced by intraamniotic infusions of interleukin-1beta and tumor necrosis factor-alpha but not by interleukin-6 or interleukin-8 in a nonhuman primate model. Am J Obstet Gynecol 195(6): 1578-1589. doi:10.1016/j.ajog.2006.06.072. PubMed: 17132473. 17132473

[28] Håland G, Carlsen KC, Sandvik L, Devulapalli CS, Munthe-Kaas MC et al. (2006) Reduced lung function at birth and the risk of asthma at 10 years of age. N Engl J Med 355(16): 1682-1689. doi:10.1056/NEJMoa052885. PubMed: 17050892. 17050892

[29] Martinez FD (2009) The origins of asthma and chronic obstructive pulmonary disease in early life. Proc Am Thorac Soc 6(3): 272-277. doi:10.1513/pats.200808-092RM. PubMed: 19387029. 19387029 PMC2677402

[30] Stern DA, Morgan WJ, Wright AL, Guerra S, Martinez FD (2007) Poor airway function in early infancy and lung function by age 22 years: a non-selective longitudinal cohort study. Lancet 370(9589): 758-764. doi:10.1016/S0140-6736(07)61379-8. PubMed: 17765525. 17765525 PMC2831283

[31] Vonk JM, Boezen HM, Postma DS, Schouten JP, Van Aalderen WM et al. (2004) Perinatal risk factors for bronchial hyperresponsiveness and atopy after a follow-up of 20 years. J Allergy Clin Immunol 114(2): 270-276. doi:10.1016/j.jaci.2004.03.051. PubMed: 15316502. 15316502

[32] World Health Organization (2007) Global surveillance, prevention and control of chronic respiratory diseases: A comprehensive approach. Bousquet J Khaltaev N Switzerland pp. 1-146.

[33] Prendergast M, May C, Broughton S, Pollina E, Milner AD et al. (2011) Chorioamnionitis, lung function and bronchopulmonary dysplasia in prematurely born infants. Arch Dis Child Fetal Neonatal Ed 96(4): F270-F274. doi:10.1136/adc.2010.189480. PubMed: 21097839. 21097839

[34] Gravett MG, Novy MJ, Rosenfeld RG, Reddy AP, Jacob T et al. (2004) Diagnosis of intra-amniotic infection by proteomic profiling and identification of novel biomarkers. JAMA 292(4): 462-469. doi:10.1001/jama.292.4.462. PubMed: 15280344. 15280344

[35] Houben ML, Bont L, Wilbrink B, Belderbos ME, Kimpen JLL et al. (2011) Clinical prediction rule for RSV bronchiolitis in healthy newborns: prognostic birth cohort study. Pediatrics 127(1): 35-41. doi:10.1542/peds.2010-0581. PubMed: 21187309. 21187309

[36] Simoes EA, Carbonell-Estrany X, Fullarton JR, Liese JG, Figueras-Aloy J et al. (2008) A predictive model for respiratory syncytial virus (RSV) hospitalisation of premature infants born at 33-35 weeks of gestational age, based on data from the Spanish FLIP study. Respir Res 9(1): 78. doi:10.1186/1465-9921-9-78. 19063742 PMC2636782

[37] DeVincenzo J, Lambkin-Williams R, Wilkinson T, Cehelsky J, Nochur S et al. (2010) A randomized, double-blind, placebo-controlled study of an RNAi-based therapy directed against respiratory syncytial virus. Proc Natl Acad Sci U S A 107(19): 8800-8805. doi:10.1073/pnas.0912186107. PubMed: 20421463. 20421463 PMC2889365

[38] Olszewska W, Openshaw P (2009) Emerging drugs for respiratory syncytial virus infection. Expert Opin Emerg Drugs 14(2): 207-217. doi:10.1517/14728210902946399. PubMed: 19453286. 19453286 PMC2705842

