# Supplementary figures and images for: Soluble Leukocyte-Associated Ig-Like Receptor-1 in Amniotic Fluid Is of Fetal Origin and Positively Associates with Lung Compliance
Source: PLoS One. 2013 Dec 26;8(12):e83920. doi: 10.1371/journal.pone.0083920 (PMC3873398; doi:10.1371/journal.pone.0083920)

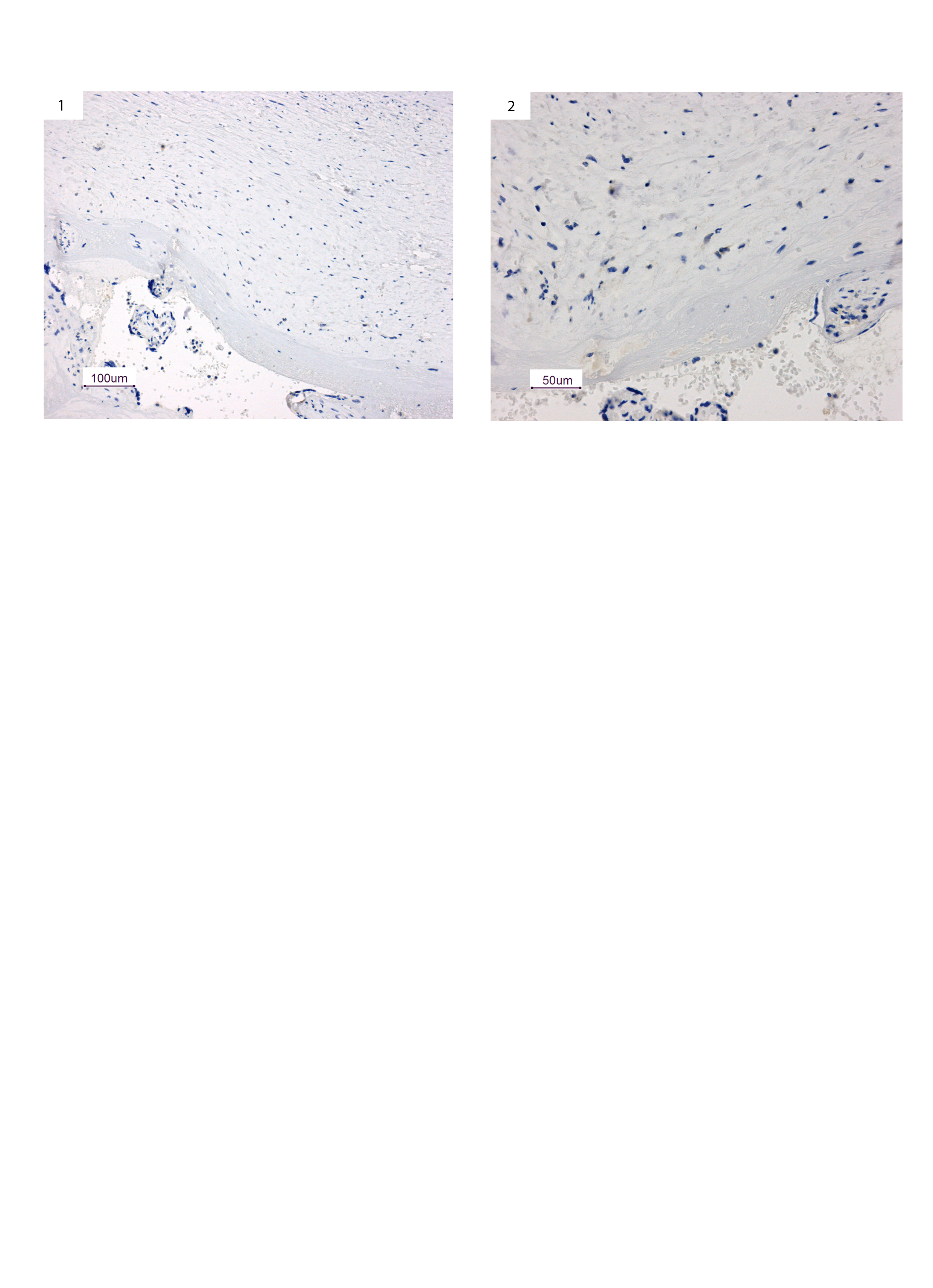

Supplement: Figure S1 — No LAIR-1 positive cells present in placenta tissue during spontaneous onset of labor at term: Placenta without histological signs of chorioamnionitis. Immunohistology of placenta. Standard H/E staining was applied to samples without immunohistology staining. LAIR-1 positive cells are absent in the chorionic plate (1+2), the umbilical cord, and the chorionic membranes (data not shown). (ZIP) [file pone.0083920.s001.zip › Figure S1.tif]

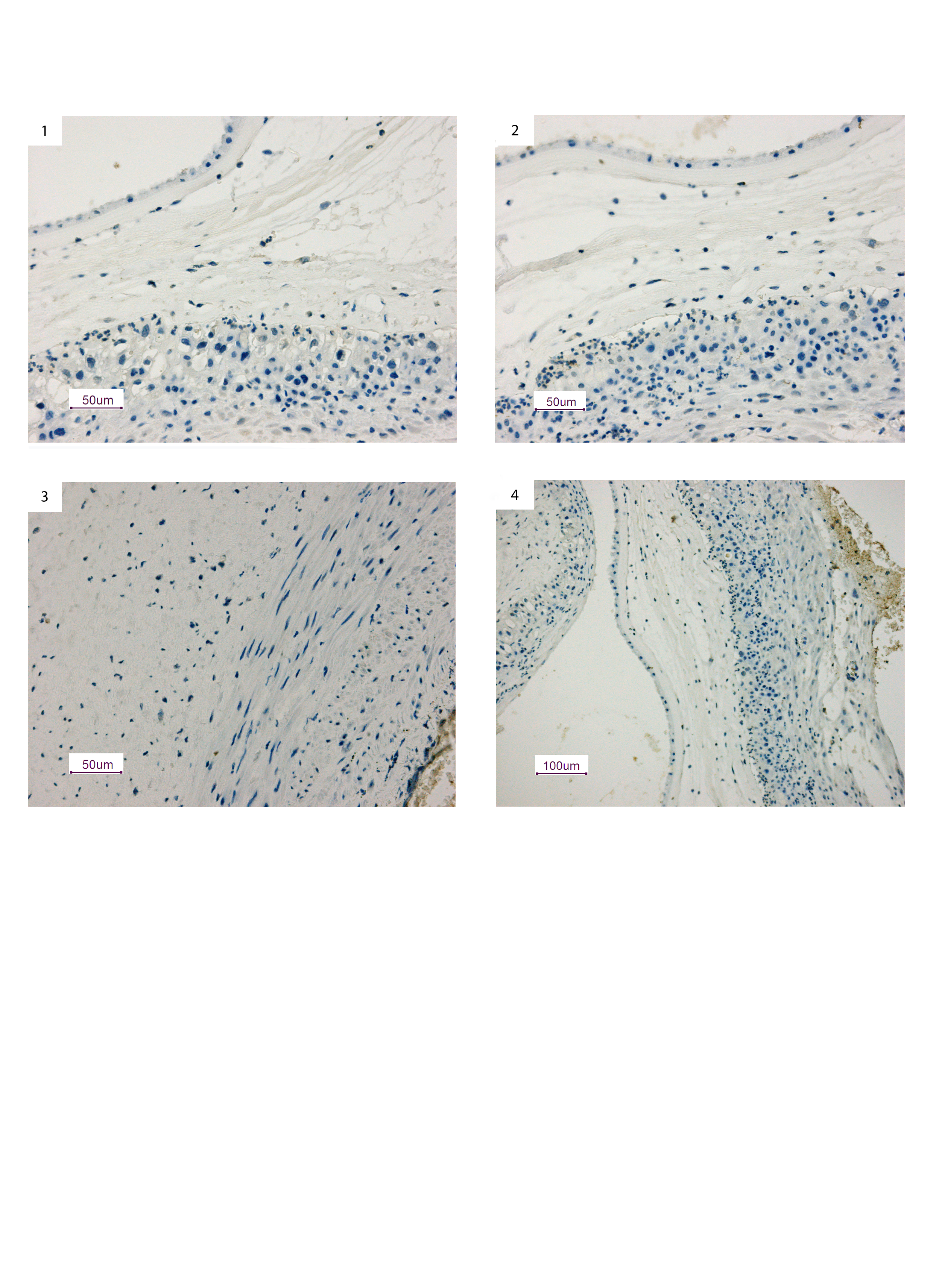

Supplement: Figure S2 — No LAIR-1 positive cells present in placenta tissue during spontaneous onset of labor at term: Placenta with mild signs of chorioamnionitis. Immunohistology of placenta. Standard H/E staining was applied to samples without immunohistology staining. LAIR-1 positive cells are sparsely present in the chorionic membranes (1+2+4), the stromal cells of Wharton’s jelly of the umbilical cord (3), and the chorionic plate (data not shown). (ZIP) [file pone.0083920.s002.zip › Figure S2.tif]

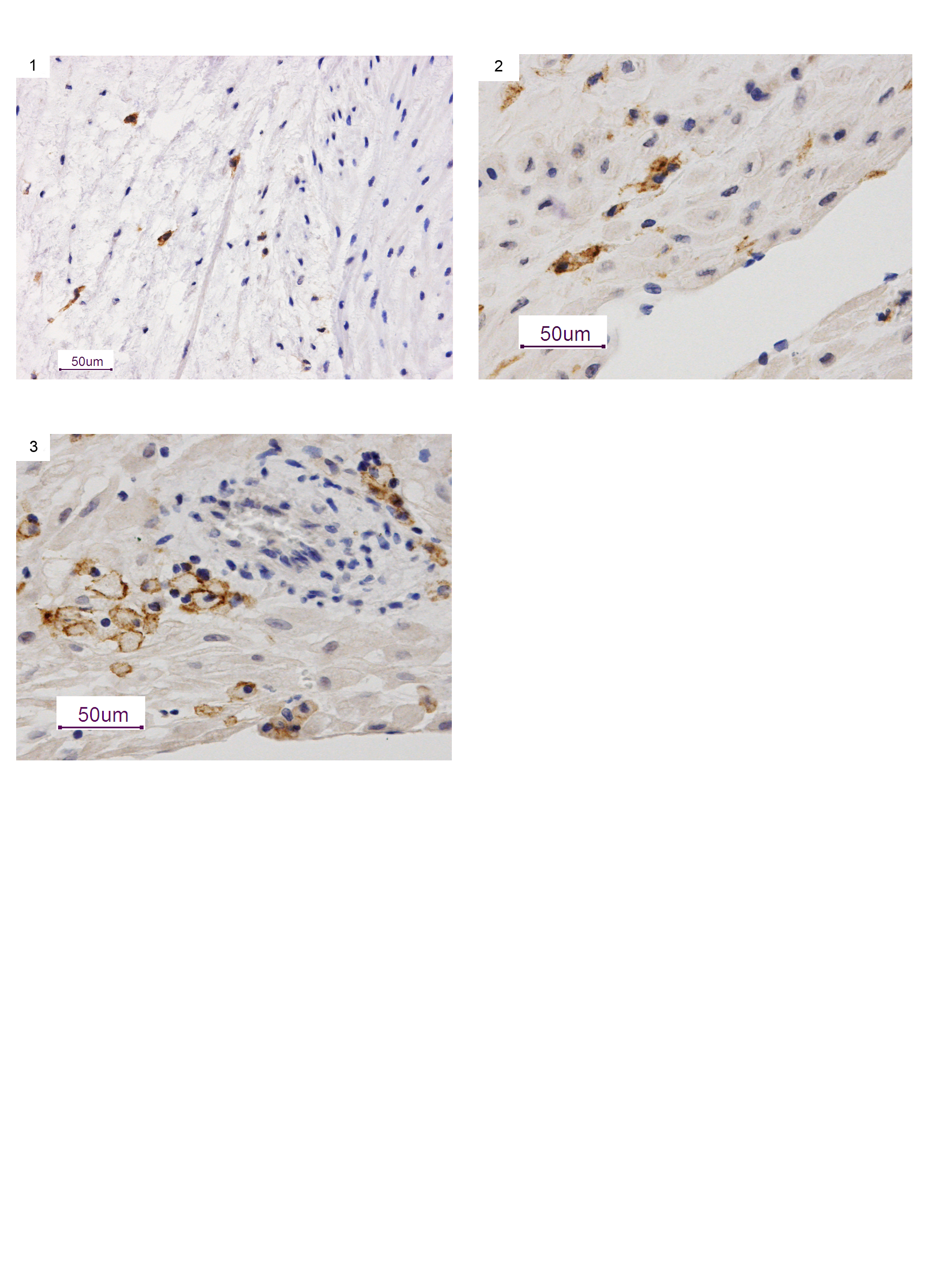

Supplement: Figure S3 — No LAIR-1 positive cells present in placenta tissue during spontaneous onset of labor at term: Placenta with severe signs of chorioamnionitis. Immunohistology of placenta. Standard H/E staining was applied to samples without immunohistology staining. LAIR-1 positive cells are infrequently present in the chorionic plate (see Figure 2E), the stromal cells of Wharton’s jelly of the umbilical cord (1), and the maternal side of the chorionic membranes (2+3). (ZIP) [file pone.0083920.s003.zip › Figure S3.tif]

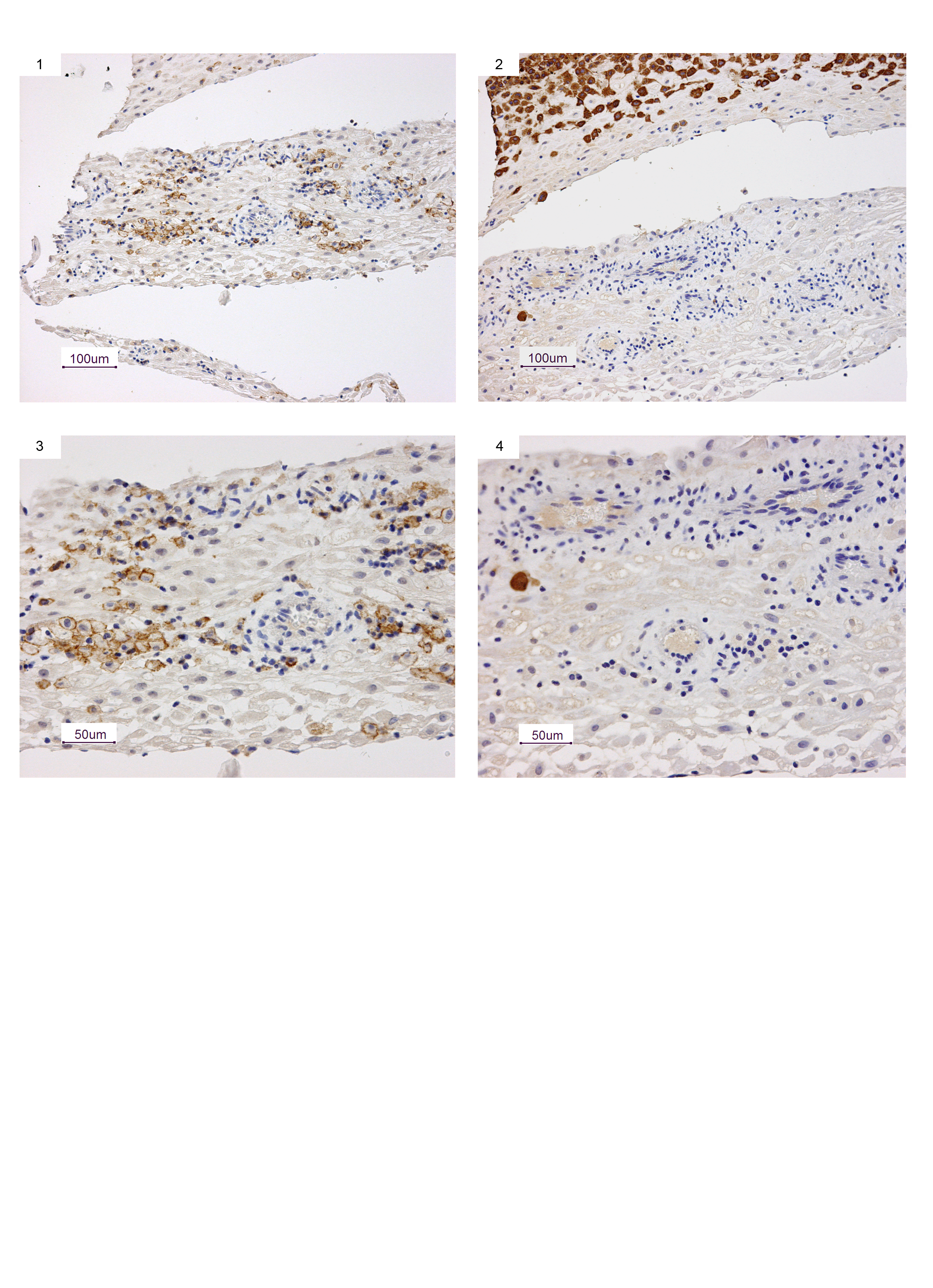

Supplement: Figure S4 — No LAIR-1 positive cells present in placenta tissue during spontaneous onset of labor at term: Placenta with severe signs of chorioamnionitis. Immunohistology of placenta. Standard H/E staining was applied to samples without immunohistology staining. LAIR-1 (left, 1+3) and keratin (right, 2+4) immunohistology. LAIR-1 positive cells are present in keratin negative regions (decidua) of the chorionic membranes. On these mononuclear cells, LAIR-1 staining is predominantly found on the cell membrane. CD68 staining (macrophage marker) of these cells is negative (data not shown). LAIR-1 positive cells are sparsely present in keratin positive regions (trophoblast) of chorionic membranes. (ZIP) [file pone.0083920.s004.zip › Figure S4.tif]

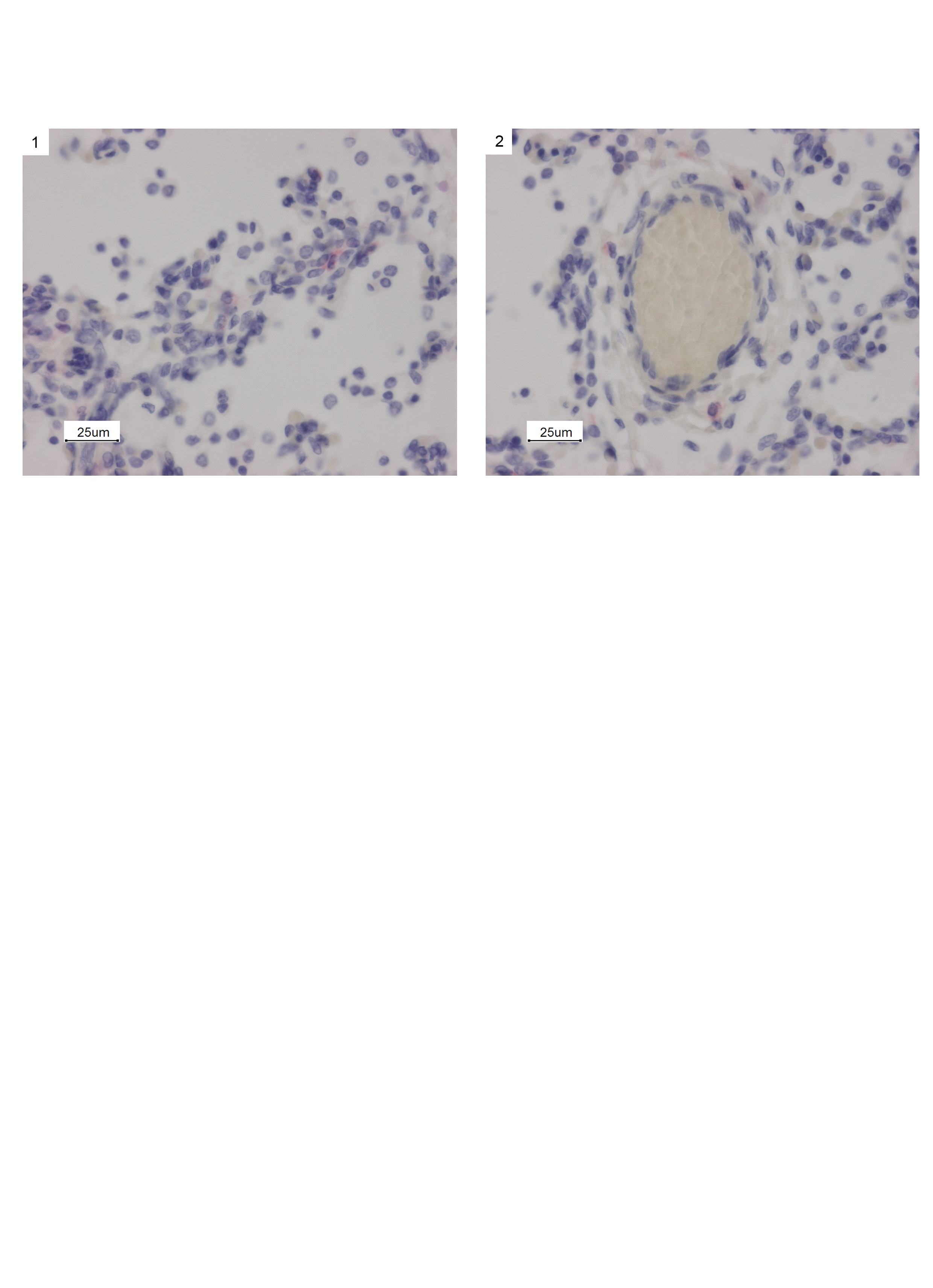

Supplement: Figure S5 — No LAIR-1 positive epithelial cells present in lung tissue of second term fatal neonatal case. Immunohistology of lung tissue of a child (second case) that died perinatally without evidence of any pulmonary disorder. Lung microscopy without abnormalities, except for weak positive LAIR-1 staining interstitially (1+2). (ZIP) [file pone.0083920.s005.zip › Figure S5.tif]

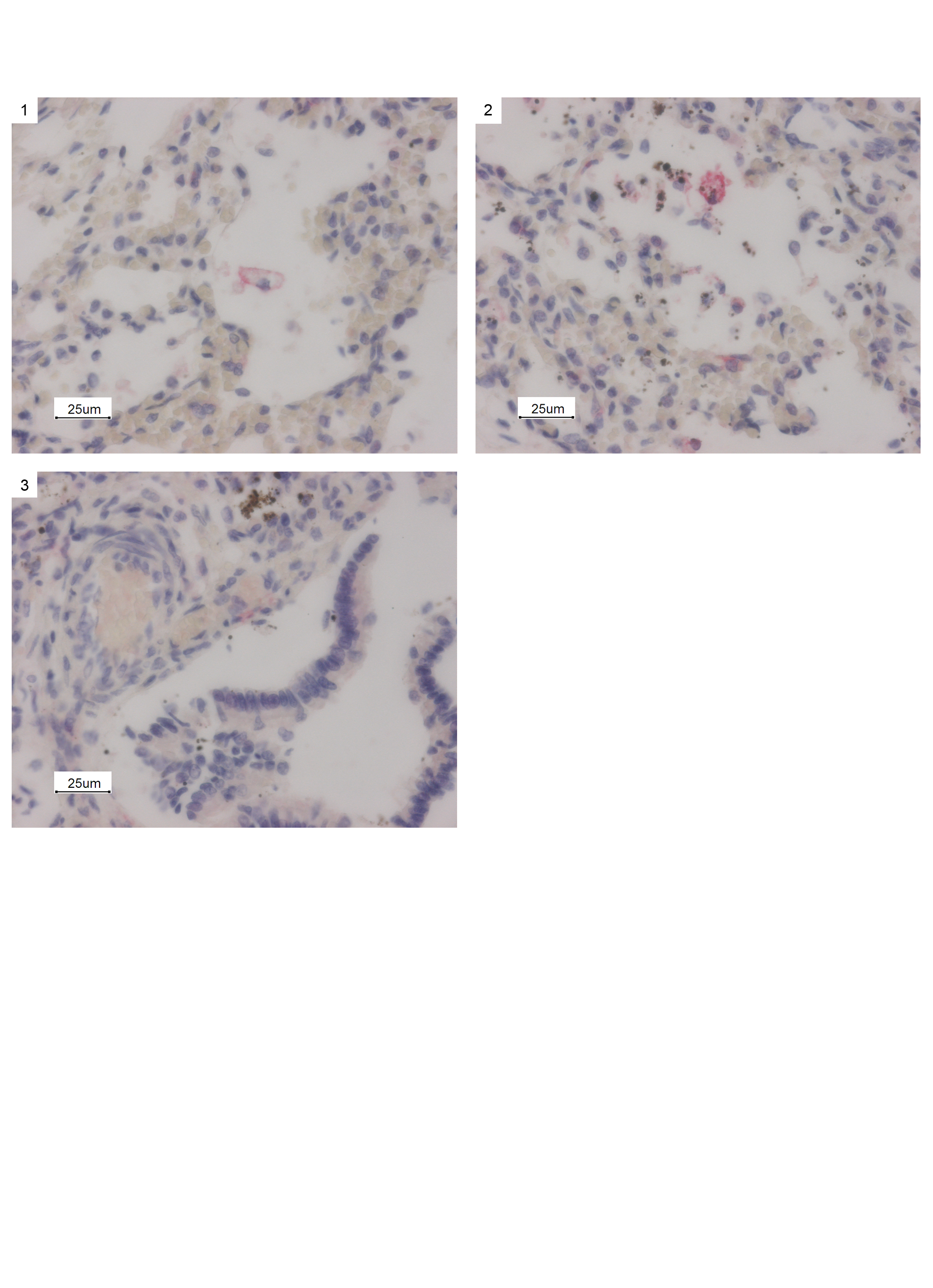

Supplement: Figure S6 — No LAIR-1 positive epithelial cells present in lung tissue of third term fatal neonatal case. Immunohistology of lung tissue of a child (third case) that died perinatally without evidence of any pulmonary disorder. Lung microscopy without signs of inflammation and negative LAIR-1 staining, except for alveolar macrophages (1-3). (ZIP) [file pone.0083920.s006.zip › Figure S6.tif]

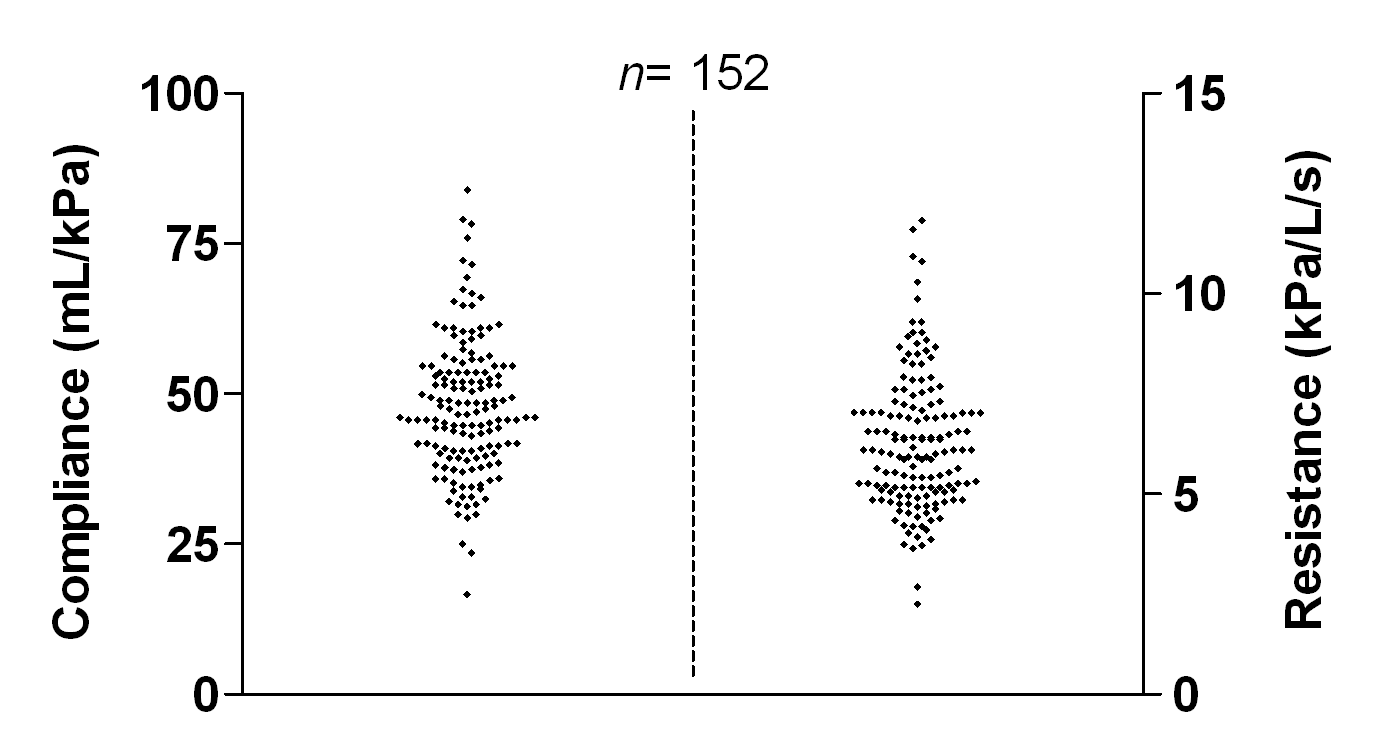

Supplement: Figure S7 — Compliance and resistance of the respiratory system of healthy term newborns. Newborn lung compliance (left Y-axis) and resistance (right Y-axis) were assessed using the single occlusion technique during physiologic sleep. Mean (standard deviation) compliance 47.8 (11.3) mL/kPa. Mean (standard deviation) resistance 6.27 (1.7) kPa/L/s. (ZIP) [file pone.0083920.s007.zip › Figure S7.tif]
