# Supplementary material for: Soluble Leukocyte-Associated Ig-Like Receptor-1 in Amniotic Fluid Is of Fetal Origin and Positively Associates with Lung Compliance
Source: PLoS One. 2013 Dec 26;8(12):e83920. doi: 10.1371/journal.pone.0083920 (PMC3873398; doi:10.1371/journal.pone.0083920)
Supplement: Table S1 — Baseline characteristics. Values represent mean (SD) or percentage. P-values for Student’s T test or X2 test. Missing values: parental atopy and / or asthma n=7 (2%), maternal antepartum smoking n=13 (3%). NA denotes not applicable. (DOC) [file pone.0083920.s008.doc]

| **Table S1. Baseline characteristics** | | | | |
| --- | --- | --- | --- | --- |
|  | Lung function measurement | | |  |
|  | Total  *n*=372 | Passed  *n*=152 | Failed  *n*=220 | *P*-value |
| *Delivery and birth* | | | | |
| Gestational age (wk) | 40.0 (1.0) | 40.1 (1.1) | 39.9 (1.0) | .07 |
| Birth weight (kg) | 3.61 (0.47) | 3.65 (0.47) | 3.58 (0.46) | .17 |
| Male gender | 51% | 52% | 51% | .77 |
| Antepartum maternal smoking | 10% | 10% | 11% | .90 |
| Parental asthma | 23% | 24% | 23% | .83 |
| Parental atopy | 56% | 54% | 57% | .61 |
