# Supplementary material for: Soluble Leukocyte-Associated Ig-Like Receptor-1 in Amniotic Fluid Is of Fetal Origin and Positively Associates with Lung Compliance
Source: PLoS One. 2013 Dec 26;8(12):e83920. doi: 10.1371/journal.pone.0083920 (PMC3873398; doi:10.1371/journal.pone.0083920)
Supplement: Table S2 — Amniotic fluid cytokine and chemokine correlation matrix. Spearman’s ρ (upper) and P-value (lower). Sample sizes were 40-42. (DOC) [file pone.0083920.s009.doc]

**Table S**2. Amniotic fluid cytokine and chemokine correlation matrix

|  | IL6 | IL8 | TNFα | IL1β | IL5 | IL10 | IL12p70 | IL17 | IL18 | MCP1 | MIF | sICAM | MIP1α | Eotaxin | IP10 | MIG | IL23 |
| --- | --- | --- | --- | --- | --- | --- | --- | --- | --- | --- | --- | --- | --- | --- | --- | --- | --- |
| sLAIR-1 | -0,07 | -0,04 | 0,21 | 0,22 | 0,21 | 0,03 | 0,10 | 0,06 | 0,07 | -0,03 | 0,10 | 0,24 | 0,02 | -0,19 | -0,29 | -0,10 | 0,07 |
|  | NS | NS | NS | NS | NS | NS | NS | NS | NS | NS | NS | NS | NS | NS | NS | NS | NS |
| IL-6 |  | 0,73 | 0,51 | 0,55 | 0,25 | 0,72 | 0,16 | 0,30 | 0,62 | 0,40 | 0,05 | 0,30 | 0,62 | 0,50 | 0,05 | 0,22 | 0,33 |
|  |  | **<0,01** | **<0,01** | **<0,01** | NS | **<0,01** | NS | NS | **<0,01** | **0,01** | NS | NS | **<0,01** | **<0,01** | NS | NS | **0,04** |
| IL-8 |  |  | 0,64 | 0,69 | 0,34 | 0,58 | 0,23 | 0,43 | 0,52 | 0,54 | 0,38 | 0,32 | 0,61 | 0,46 | 0,05 | 0,40 | 0,45 |
|  |  |  | **<0,01** | **<0,01** | **0,03** | **<0,01** | NS | **0,01** | **<0,01** | **<0,01** | **0,02** | **0,045** | **<0,01** | **<0,01** | NS | **0,01** | **<0,01** |
| TNF-α |  |  |  | 0,69 | 0,33 | 0,63 | 0,24 | 0,35 | 0,37 | 0,44 | 0,48 | 0,43 | 0,61 | 0,39 | 0,01 | 0,22 | 0,43 |
|  |  |  |  | **<0,01** | **0,04** | **<0,01** | NS | **0,03** | **0,02** | **<0,01** | **<0,01** | **0,01** | **<0,01** | **0,01** | NS | NS | **0,01** |
| IL-1β |  |  |  |  | 0,36 | 0,60 | 0,60 | 0,51 | 0,60 | 0,42 | 0,52 | 0,42 | 0,62 | 0,34 | 0,02 | 0,35 | 0,43 |
|  |  |  |  |  | **0,02** | **<0,01** | **<0,01** | **<0,01** | **<0,01** | **0,01** | **<0,01** | **0,01** | **<0,01** | **0,03** | NS | **0,03** | **0,01** |
| IL-5 |  |  |  |  |  | 0,65 | 0,49 | 0,73 | 0,16 | 0,60 | 0,34 | 0,52 | 0,52 | 0,56 | 0,29 | 0,57 | 0,83 |
|  |  |  |  |  |  | **<0,01** | **<0,01** | **<0,01** | NS | **<0,01** | **0,03** | **<0,01** | **<0,01** | **<0,01** | **0,07** | **<0,01** | **<0,01** |
| IL-10 |  |  |  |  |  |  | 0,56 | 0,74 | 0,49 | 0,73 | 0,30 | 0,53 | 0,87 | 0,78 | 0,25 | 0,57 | 0,81 |
|  |  |  |  |  |  |  | **<0,01** | **<0,01** | **<0,01** | **<0,01** | NS | **<0,01** | **<0,01** | **<0,01** | NS | **<0,01** | **<0,01** |
| IL-12p70 |  |  |  |  |  |  |  | 0,76 | 0,42 | 0,42 | 0,42 | 0,43 | 0,50 | 0,49 | 0,21 | 0,43 | 0,63 |
|  |  |  |  |  |  |  |  | **<0,01** | **0,01** | **0,01** | **0,01** | **0,01** | **<0,01** | **<0,01** | NS | **0,01** | **<0,01** |
| IL-17 |  |  |  |  |  |  |  |  | 0,40 | 0,66 | 0,39 | 0,54 | 0,68 | 0,60 | 0,32 | 0,63 | 0,89 |
|  |  |  |  |  |  |  |  |  | **0,01** | **<0,01** | **0,01** | **<0,01** | **<0,01** | **<0,01** | **0,048** | **<0,01** | **<0,01** |
| IL-18 |  |  |  |  |  |  |  |  |  | 0,17 | 0,02 | 0,14 | 0,32 | 0,24 | -0,01 | 0,19 | 0,29 |
|  |  |  |  |  |  |  |  |  |  | NS | NS | NS | **0,045** | NS | NS | NS | NS |
| MCP-1 |  |  |  |  |  |  |  |  |  |  | 0,49 | 0,37 | 0,78 | 0,84 | 0,41 | 0,65 | 0,70 |
|  |  |  |  |  |  |  |  |  |  |  | **<0,01** | **0,02** | **<0,01** | **<0,01** | **0,01** | **<0,01** | **<0,01** |
| MIF |  |  |  |  |  |  |  |  |  |  |  | 0,30 | 0,41 | 0,33 | 0,04 | 0,15 | 0,41 |
|  |  |  |  |  |  |  |  |  |  |  |  | NS | **0,01** | **0,04** | NS | NS | **0,01** |
| sICAM |  |  |  |  |  |  |  |  |  |  |  |  | 0,45 | 0,35 | 0,06 | 0,26 | 0,58 |
|  |  |  |  |  |  |  |  |  |  |  |  |  | **<0,01** | **0,02** | NS | NS | **<0,01** |
| MIP-1α |  |  |  |  |  |  |  |  |  |  |  |  |  | 0,81 | 0,35 | 0,56 | 0,68 |
|  |  |  |  |  |  |  |  |  |  |  |  |  |  | **<0,01** | **0,03** | **<0,01** | **<0,01** |
| Eotaxin |  |  |  |  |  |  |  |  |  |  |  |  |  |  | 0,48 | 0,56 | 0,69 |
|  |  |  |  |  |  |  |  |  |  |  |  |  |  |  | **<0,01** | **<0,01** | **<0,01** |
| IP10 |  |  |  |  |  |  |  |  |  |  |  |  |  |  |  | 0,68 | 0,25 |
|  |  |  |  |  |  |  |  |  |  |  |  |  |  |  |  | **<0,01** | NS |
| MIG |  |  |  |  |  |  |  |  |  |  |  |  |  |  |  |  | 0,62 |
|  |  |  |  |  |  |  |  |  |  |  |  |  |  |  |  |  | **<0,01** |
